# Intake of arachidonic acid-containing lipids in adult humans: dietary surveys and clinical trials

**DOI:** 10.1186/s12944-019-1039-y

**Published:** 2019-04-16

**Authors:** Hiroshi Kawashima

**Affiliations:** Research Institute, Suntory Global Innovation Center Ltd., 8-1-1 Seikadai, Seika, Kyoto 619-0284 Japan

**Keywords:** Arachidonic acid, Dietary survey, Docosahexaenoic acid, Eicosapentaenoic acid, Long-chain polyunsaturated fatty acid

## Abstract

Long-chain polyunsaturated fatty acids (LCPUFAs) have important roles in physiological homeostasis. Numerous studies have provided extensive information about the roles of n-3 LCPUFA, such as docosahexaenoic acid and eicosapentaenoic acid. Arachidonic acid (ARA) is one of the major n-6 LCPUFAs and its biological aspects have been well studied. However, nutritional information for ARA is limited, especially in adult humans. This review presents a framework of dietary ARA intake and the effects of ARA supplementation on LCPUFA metabolism in adult humans, and the nutritional significance of ARA and LCPUFA is discussed.

## Background

**Fig. 1:**
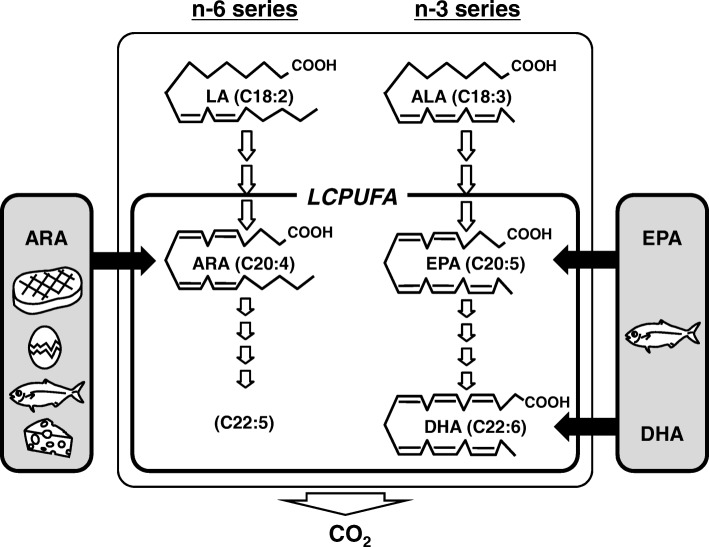
Scheme of long-chain polyunsaturated fatty acid (LCPUFA) metabolism. LCPUFA in the body has two origins. One is the direct incorporation from dietary animal foods, and the other is the biosynthesis from n-6 or n-3 precursor PUFA, linoleic acid (LA) or α–linolenic acid (ALA), respectively. All the fatty acids including LCPUFA are mainly metabolized to CO_2_ by β–oxidation and excreted in the breath

Long-chain polyunsaturated fatty acids (LCPUFAs) are the main constituents of biomembranes and have important roles in physiological homeostasis. LCPUFAs consist of two individual series, namely, n-6 and n-3 series. Humans cannot synthesize n-6 and n-3 PUFAs de novo, and convert linoleic acid (LA) and alpha-linolenic acid (ALA) obtained from foods to n-6 and n-3 LCPUFAs, respectively. LCPUFAs in the body are consequently derived from both the conversion of LA or ALA and the direct intake of respective LCPUFAs (Fig. 1). The major n-6 LCPUFA is arachidonic acid (ARA), and the major n-3 LCPUFAs are docosahexaenoic acid (DHA) and eicosapentaenoic acid (EPA). The importance of dietary intake of DHA and EPA has been extensively studied [1–3], but there is limited information for n-6 LCPUFA. Studies of ARA have focused on biological aspects, and many lipid mediators from ARA have been discovered and contribute to its medical application [4–9]. However, little attention has been paid to the dietary intake and clinical effects of ARA itself in adult humans [10], although the knowledge in infant nutrition has progressed exceptionally [11, 12]. Recently, the efficacy of ARA supplementation has been reported in the fields of cognitive attention and memory [13–15], mood states [16], coronary circulation [17] and cirrhosis [18, 19]; and further nutritional understanding of ARA intake is expected.

The aim of this review is to provide an overview of the impact of ARA intake in adult humans. The author outlines the dietary intake of ARA from daily foods in adult humans of various countries, and reviews clinical trials of supplementation of ARA-containing lipids.

## Food sources of ARA

ARA is found only in animal-derived foods because plants cannot synthesize C-20 LCPUFAs. The main food sources of ARA are meat, poultry, eggs, fish and dairy foods, as shown in Table 1 [20, 21]. ARA is contained in most animal foods [22, 23]; however, the contents of ARA are moderate, < 200 mg per 100 g of these foods, revealing the wide but small distribution of ARA in major animal foods. This is in stark contrast to the case of DHA/EPA. DHA/EPA is only found in seafood, however the content of DHA/EPA reaches from several hundred mg to more than 1 g per 100 g of fish. These data suggest that ARA intake may fluctuate less with the intake of certain animal food groups, in contrast to the case of DHA/EPA in fish.

**Table 1:** Content of ARA and the other fatty acids per 100 g edible portion of animal foods

Food group	Ref.^a^	Total fat (mg)	Fatty acids (mg)^b^					
			PA	OA	LA	ARA	EPA	DHA
Meats and poultry								
Pork, loin, whole, lean and fat, raw	C	12,580	2720	5140	1110	80	0	0
Pork, medium type breed, loin, lean and fat, raw	J	22,600	5600	9100	1900	68	0	12
Chicken, broiler, thigh, meat and skin, raw	C	16,610	3511	5832	3096	104	4	7
Chicken, broiler, thigh, meat with skin, raw	J	14,200	3300	5800	1600	79	1	7
Beef, hip, inside (top) round steak, boneless, lean, raw	C	2210	520	910	120	40	0	0
Beef, inside round, lean, raw	J	4300	890	1500	120	24	4	1
Eggs								
Chicken, whole, fresh or frozen, raw	C	10,010	2218	3810	1109	156	2	72
Hen, whole, raw	J	10,300	2100	3500	1300	150	0	120
Fishes and seafoods								
Salmon, pink (humpback), raw	C	6700	1044	1108	102	127	547	859
Pink salmon, raw	J	6600	790	920	81	31	400	690
Flatfish (flounder or sole or plaice), raw	C	1930	282	358	45	30	137	108
Righteye flounder, brown sole, raw	J	1300	150	140	10	50	100	72
Sardine, pacific, canned in tomato sauce, drained with bones	C	10,450	1738	1851	123	190	532	864
Sardine, Japanese pilchard, canned products, in tomato sauce	J	10,800	1900	1200	140	160	1300	1100
Milk and dairy products								
Cheese, cream	C	34,240	8497	7923	1032	50	0	0
Cheese, cream	J	33,000	8700	6400	570	38	20	6

^a^C, Canadian nutrient file version 2015 [20]; J, Standard tables of food composition in Japan 2015 (seventh revised edition) [21]

^b^*PA* palmitic acid, *OA* oleic acid, *LA* linoleic acid, *ARA* arachidonic acid, *EPA* eicosapentaenoic acid, *DHA* docosahexaenoic acid

Table 2 shows the contribution of each food to ARA intake [24–28]. The proportion of meat and poultry is high (43–62%) in Europe [24, 25] and the United States [26], but is only 20–30% in Japan [27] and Korea [28]. The contribution of eggs is high in Japan. Fish and seafood, the main sources of DHA/EPA, are also significant sources of ARA (4.9–12.2%) in all the countries. In elderly Japanese, the contribution of fish to ARA intake reached approximately 30% and was equal to that of meat [29]. It is equivocal that foods of plant origin are described as contributors to ARA intake in some studies (potato, rice and pasta, 7.1% [25]; nuts. 9% [26]), as these plants cannot synthesize ARA or C-20 LCPUFAs. This suggests that the qualitative or quantitative accuracy of ARA content in food composition tables is not always complete, which may be one of the reasons why the calculation of ARA intake seems inaccurate in some cases, as described below.

**Table 2:** Food sources of ARA (% of the total ARA intake)^a^

Food group	France [24]	UK [25]	USA [26]	Japan [27]	Korea [28]
Meats and poultry	50.3	62.3	43	22.5	28.4
Eggs	16.9	11.1	19	47.2	17.9
Fishes and seafoods	11.1	4.9	9	11.1	12.2
Milk and dairy products	1.1	nd^b^	nd	3.0	14.3
Sweet product	11.6	nd	nd	nd	nd
Plant origin					
Cereals, fruit and vegetable	2.9	nd	nd	nd	nd
Potato, rice and pasta	nd	7.1	nd	nd	nd
Nuts	0.0	nd	9	nd	nd

Total of each percentage does not reach 100% due to lack of the minor contributors

^a^Original data are classified to the nearest food group

^b^*nd* not described

## Dietary intake of ARA

Dietary intakes of LCPUFA in 175 countries were estimated using food balance sheets from the Food and Agriculture Organization and food composition tables [30]. The calculated ARA intakes ranged from 101 to 351 mg/day in advanced countries, and 44–331 mg/day in developing countries. This is a useful calculation derived from the statistical data of international agriculture and trade; however, it is only an estimation for individual countries and is not based on accurate amounts of LCPUFAs derived from direct measurements of food consumption of individuals or specific groups. The author therefore reviewed the studies to investigate the amount of dietary ARA using nutritional survey methods.

Table 3 shows data compiled from surveys of more than 1000 healthy adults in a study and published from January 2001 [24, 25, 31–41]. The data were obtained from various areas, i.e., Europe, North America, Africa, Asia and Oceania. The amounts of dietary ARA intake range widely from 9 to 290 mg/day. The large differences may be attributable to the survey method or the dietary habits in individual countries. First, with respect to the survey methods, it is notable that similar amounts of ARA intake were reported in four studies [24, 32, 38, 40] using dietary record (DR) or 24-h diet recall (169–230 (male) and 117–160 (female) mg/day). Generally, the quantitative accuracy of DR or 24-h recall is thought to be superior to that of the food frequency questionnaire (FFQ). Most of the other studies using DR or 24-h recall with smaller numbers of participants also reported that ARA intakes were around 100 mg/day or more, although there are some exceptions (Table 4) [28, 42–48]. These studies suggest that ARA intake, at least in advanced countries, is 100–250 mg/day for normal healthy adults. This is a similar but narrower range compared to the calculation from the statistical data described above [30]. ARA intake in the tens of mg per day reported in some surveys is similar to or less than that of American vegetarians (3–44 mg/day) [37], and seems too low. Similar results were reported in the other studies with limited numbers of participants in Germany [49], Norway [50], Canada [51, 52] and Japan [27, 29, 53, 54]. Studies reporting that ARA intake is several mg/day are likely to contain errors in their calculation methods. To accurately assess the amount of ARA intake, it may be important to reexamine and revise the ARA content reported in various food composition tables.

**Table 3:** Dietary survey of intake of ARA, EPA and DHA in adult humans (> 1000 participants in a study and from January 2001)

Country	Participant				Dietary survey^c^	LCPUFA intake (mg/day)^d^			Ref.
	Sex^a^	Age (y)^b^	Other classification	N		ARA	EPA	DHA	
Europe									
Finland	M&F	30–49	–	1212	FFQ	95 ± 0.84^e^	160 ± 3.1^e^	420 ± 8.7^e^	[31]
		50-79	–	980		97 ± 1.1^e^	190 ± 4.6^e^	510 ± 13^e^	
France	M	45–63	–	2099	ten 24-h DR	204 ± 66	150 ± 112	273 ± 191	[24]
	F	35–63	–	2785		152 ± 49	118 ± 94	226 ± 171	
Germany	M	45–65	Heidelberg	1013	24-h recall	230 ± 250	100 ± 300	190 ± 480	[32]
			Potsdam	1032		230 ± 250	130 ± 380	210 ± 490	
	F	35–64	Heidelberg	1078		160 ± 190	70 ± 230	140 ± 330	
			Potsdam	898		140 ± 160	80 ± 230	140 ± 280	
Spain	F	20–79	–	1865	FFQ	290 ± 110	220 ± 90	300 ± 120	[33]
United Kingdom	M&F	16–79	–	1455	FFQ	9^f^	290^f^	380^f^	[25]
North America									
United States	F	> 45	Health Professional	37,547	FFQ	70^f^	20^f^	60^f^	[34]
United States	M&F	> 30	–	2837	FFQ	120 ± 80	45 ± 50	82 ± 73	[35]
United States	F	< 65	–	1500	FFQ	70 ± 60	40 ± 50	90 ± 90	[36]
United States &Canada	M&F	> 30	Nonvegetarian	33,634	FFQ	84 ± 0.3^e^	nd^g^	182 ± 1.2^e^	[37]
			Semi-vegetarian	4042		27 ± 0.7^e^	nd	70 ± 3.6^e^	
			Pesco vegetarian	6583		44 ± 0.6^e^	nd	187 ± 2.8^e^	
			Lacto-ovo vegetarian	21,799		13 ± 0.3^e^	nd	34 ± 1.5^e^	
			Strict vegetarian	5694		3 ± 0.6^e^	nd	18 ± 3^e^	
Africa, Asia and Oceania									
Australia	M	> 19	–	5081	24-h recall	191 ± 2^e^	91 ± 3^e^	117 ± 5^e^	[38]
	F	> 19	–	5770		117 ± 2^e^	60 ± 2^e^	83 ± 3^e^	
China	F	40–70	–	74,943	FFQ	50^f^	nd^h^	nd^h^	[39]
Japan	M	40–49	–	241	3-day DR	179 ± 66	233 ± 211	437 ± 331	[40]
		50–59	–	268		185 ± 64	368 ± 296	662 ± 476	
		60–69	–	262		182 ± 63	403 ± 263	718 ± 422	
		70–79	–	243		171 ± 64	390 ± 257	692 ± 437	
	F	40–49	–	263		153 ± 52	217 ± 185	414 ± 305	
		50–59	–	259		148 ± 51	268 ± 202	487 ± 322	
		60–69	–	261		149 ± 53	300 ± 196	532 ± 312	
		70–79	–	245		144 ± 55	300 ± 219	525 ± 340	
South Africa	M	> 35	Rural	333	FFQ	34^f^	38^f^	62^f^	[41]
			Urban	393		102^f^	61^f^	101^f^	
	F	> 35	Rural	633		33^f^	33^f^	52^f^	
			Urban	591		94^f^	46^f^	83^f^	

^a^*M* male, *F* female

^b^Mean or range

^c^*FFQ* food frequency questionnaire, *DR* diet record

^d^Data are the mean ± SD without annotation. Original data are rounded to nearest mg

^e^Mean ± SE

^f^Median

^g^*nd* not described

^h^Median of (EPA + DHA) is 70 mg/d

**Table 4:** Dietary survey of intake of ARA, EPA and DHA in adult humans (< 1000 participants by DR or 24-h recall)

Country	Participant				Dietary Survey^c^	LCPUFA intake (mg/day)^d^			Ref.
	Sex^a^	Age (y)^b^	Other classification	N		ARA	EPA	DHA	
Bangladeshi	F	16–50	Mothers of children 2–4 y	455	24-h recall	40	30	30	[42]
Belgium	F	18–39	–	641	2-day DR	56 ± 47	78 ± 156	131 ± 247	[43]
Brazil	F	18–35	Pregnant women	41	24-h recall	90	0.2	20	[44]
China	F	27.0	Changzhou area	82	7-day DR	110 ± 40	50 ± 40	40 ± 60	[45]
		27.8	Wenzhou area	20		140 ± 60	120 ± 130	180 ± 230	
Japan	F	40–49	Spring season	71	7-day DR	134 ± 39	277 ± 13	755 ± 357	[46]
Korea	M	30–85	–	107	3-day DR	135 ± 161	279 ± 690	172 ± 1114	[28]
	F	30–85	–	117		99 ± 116	159 ± 271	235 ± 1479	
South Africa	F	32.8	Urban Northern Cape	83	24-h recall	97	33	54	[47]
		32.9	Urban coastal Western Cape	81		105	36	67	
		34.8	Rural Limpopo Province	85		39	8	24	
United States	M	49	Pakistan-origin	106	24-h recall	200 ± 700	30 ± 70	90 ± 20	[48]
		49	India-origin	34		160 ± 140	10 ± 10	40 ± 40	
		46	Bangladesh-origin	34		200 ± 140	200 ± 30	300 ± 400	
	F	48	Pakistan-origin	117		200 ± 100	40 ± 100	100 ± 200	
		49	India-origin	37		100 ± 100	40 ± 100	70 ± 200	
		49	Bangladesh-origin	33		200 ± 100	300 ± 500	400 ± 800	

^a^*M* male, *F* female

^b^Mean or range

^c^*FFQ* food frequency questionnaire, *DR* diet record

^d^Data are the mean ± SD or median. Original data are rounded to nearest mg

Second, with respect to dietary habits in individual countries, it is expected that ARA intake is associated with the amount of animal food intake. This is strongly suggested by the study of vegetarians, where the strictness of animal food avoidance is proportional to the decrease in ARA intake [37]. Although a similar situation may be infrequent in advanced countries, it may occur in developing countries. ARA intake was reported to be 33–34 [41] or 39 mg/day [47] in rural areas of South Africa, which is approximately one-third of that in respective urban areas. In any case, it is expected that additional high-quality nutritional data of dietary ARA intake in various countries and groups will accumulate.

## ARA source by fermentation technique

Numerous studies for infant nutrition have clarified that DHA and ARA are present in breast milk, that infants themselves have only a weak ability to synthesize DHA and ARA endogenously from ALA and LA, and that addition of DHA and ARA to infant formula is preferred for development of infants [11, 12]. Fish oil is a good source for DHA, and has been used for an ingredient of infant formula. However, as described above, the contents of ARA are moderate in common foods. Since there was no practical source for ARA, a new ARA oil with high-quality was needed. In order to obtain oil with high ARA content for addition to infant formula, a microbial fermentation oil was developed in 1987 [55, 56]. The fungus *Mortierella alpina* accumulates large amounts of ARA-containing lipids in its cells [57], and an industrial production process for it has been established [58, 59]. This oil has been used for infant formula worldwide [60]. At the same time, ARA oil is now used for adult humans, especially the elderly, making it possible to investigate the physiological roles and efficacy of ARA [61–68].

## Supplementation of ARA-containing lipids

**Fig. 2:**
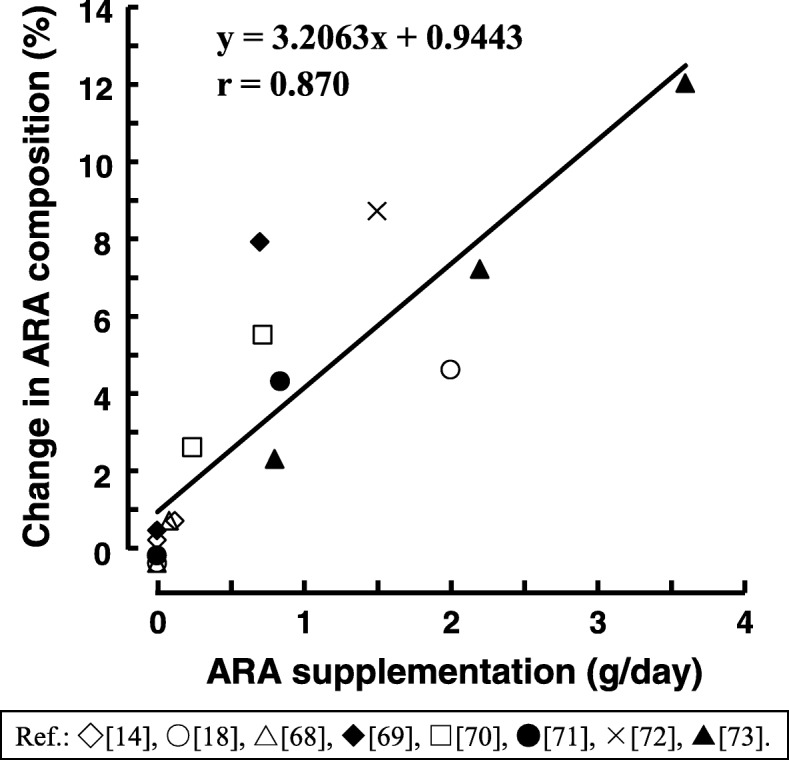
Correlation between the dose of ARA supplementation and the change of plasma ARA composition. The change of plasma ARA composition was calculated from Table 5. Neither the number of participants, supplementation period nor the existence or non-existence of DHA/EPA was taken into account. Data from individual studies are indicated with the same symbol

Table 5 summarizes the clinical trials reporting changes in ARA composition of blood in adult humans with ARA supplementation [16, 17, 19, 69–74]. The ARA-containing lipids of *M. alpina* were used for ARA supplementation in all nine studies. The conditions of the trials are different from each other. Doses of ARA as free ARA were 82.8–3600 mg/day with or without DHA/EPA. Supplementation periods were from 14 days to 3 months. Fatty acid analyses were conducted using plasma phospholipids (PL) or red blood cells (RBC). Interestingly, the smallest dose of ARA (82.8 mg/day for 3 weeks) resulted in a significant increase of ARA composition in plasma PL and RBC [69]. The second smallest dose of ARA (120 mg/day for 4 weeks) with DHA/EPA (300/100 mg/kg) also increased ARA composition of plasma PL [16]. These doses of ARA are equal to or less than the standard dietary ARA intake (100–250 mg/day), as reviewed above. These data support that dietary ARA intake from daily foods should contribute to the increase or maintenance of plasma ARA composition, which may have been understated so far. All the doses of ARA increased blood ARA levels regardless of co-supplementation with DHA/EPA. Correlation between the dose of ARA supplementation and the change of plasma ARA composition is shown in Fig. 2. The increase in plasma ARA composition is dose-dependent over a range of 82–3600 mg/kg (*r* = 0.87).

**Table 5:** Increase of ARA composition in blood by ARA supplementation to adult humans

Participant			Supplementation					Sample^c^	LCPUFA composition in blood (%)^d^						Ref.
Sex^a^	Age (y)^b^	*n*	Oil	Dose (mg/day)			Period		ARA			DHA			
				ARA	EPA	DHA			Pre	Post	Change	Pre	Post	Change	
F	18–23	23	ARA oil	82.8	0.2	0	3 weeks	Plasma PL	7.4 ± 0.8	nd^e^	0.7 ± 0.8^*^	5.6 ± 0.8	nd	− 0.5 ± 0.7	[69]
		23	Placebo	0	0	0			7.6 ± 1.1	nd	−0.4 ± 1.0	5.6 ± 1.0	nd	−0.5 ± 0.8	
		23	ARA oil	82.8	0.2	0		RBC	10.2 ± 0.8	nd	1.1 ± 0.4^*^	6.4 ± 0.5	nd	0.1 ± 0.4	
		23	Placebo	0	0	0			10.5 ± 0.8	nd	0.5 ± 0.3	6.5 ± 0.6	nd	0.0 ± 0.3	
M	55–64	51	ARA oil + fish oil	120	100	300	4 weeks	Plasma PL	8.6 ± 0.2	9.3 ± 0.2^#^	0.7 ± 0.1^*^	7.0 ± 0.2	7.8 ± 0.2^#^	0.8 ± 0.2^*^	[16]
		49	Placebo	0	0	0			8.9 ± 0.2	9.1 ± 0.2	0.2 ± 0.1	6.9 ± 0.2	7.2 ± 0.2	0.2 ± 0.1	
M&F	65 ± 3	13	ARA oil + fish oil	240	0	240	3 months	RBC	8.8 ± 1.5	12.5 ± 1.4^#^	nd	6.0 ± 1.7	10.4 ± 1.3^#^	nd	[17]
	65 ± 3	15	Placebo	0	0	0			10.0 ± 1.1	10.4 ± 1.2	nd	7.6 ± 2.2	8.5 ± 1.1	nd	
M&F	56–70	8	ARA oil	700	0	0	12 weeks	Plasma PL	9.3 ± 0.4^f,*#^	17.2 ± 0.5^f,*#^	nd	3.7 ± 0.3^f^	3.7 ± 0.4^f^	nd	[70]
	56–69	8	Placebo	0	0	0			8.6 ± 0.3^f^	9.0 ± 0.9^f^	nd	3.4 ± 0.4^f^	3.3 ± 0.4^f^	nd	
M	55–70	22	ARA oil	720	0	0	4 weeks	Plasma PL	8.8 ± 1.3	14.3 ± 2.1^#^	nd	nd	nd	nd	[71]
		22	ARA oil	240	0	0			8.6 ± 0.9	11.2 ± 1.5^#^	nd	nd	nd	nd	
		20	Placebo	0	0	0			nd	nd	nd	nd	nd	nd	
M	26–60	12	ARA oil	838	0	0	4 weeks	Plasma PL	9.6 ± 0.4	13.9 ± 0.4^*#^	nd	7.7 ± 0.3	7.4 ± 0.3	nd	[72]
		12	Placebo	0	0	0			9.5 ± 0.4	9.3 ± 0.4	nd	8.6 ± 0.4	8.4 ± 0.4	nd	
M	20–39	10	ARA oil	1500	0	0	50 days	Plasma PL	nd	19.0^g,*^	nd	nd	nd	nd	[73]
		10	Placebo	0	0	0			nd	10.3^g^	nd	nd	nd	nd	
M&F	67 ± 2.4	15	ARA oil	2000	0	0	8 weeks	Plasma PL	8.5 ± 0.6	13.1 ± 1.0^#^	nd	nd	nd	nd	[19]
	62 ± 2.3	15	Placebo	0	0	0			8.4 ± 0.6	8.0 ± 0.4	nd	nd	nd	nd	
	67 ± 2.4	15	ARA oil	2000	0	0		RBC	13.8 ± 1.1	14.8 ± 0.9^#^	nd	nd	nd	nd	
	62 ± 2.3	15	Placebo	0	0	0			10.3 ± 0.8	12.2 ± 0.9	nd	nd	nd	nd	
M	19–39	8	ARA oil + algal oil	3600	0	2900	14 days	Plasma PL	nd	24.7 ± 1.5^f,††††^	nd	nd	6.1 ± 0.3^f,††††^	nd	[74]
	19–39	8	ARA oil + algal oil	2200	0	1700			nd	19.9 ± 1.5^f,†††^	nd	nd	5.3 ± 0.5^f,†††^	nd	
	19–39	8	ARA oil + algal oil	800	0	600			nd	15.0 ± 1.6^f,††^	nd	nd	3.4 ± 0.4^f,††^	nd	
	19–39	8	Placebo	0	0	0			nd	12.7 ± 2.0^f,†^	nd	nd	2.2 ± 0.4^f,†^	nd	

^*^significant difference at *p* < 0.05 vs. the placebo group

^#^significant difference at *p* < 0.05 vs. the pre-value

^†,††,†††,††††^Values with different number of daggers are significantly different at *p* < 0.05

^a^*M* male, *F* female

^b^Mean ± SD or range

^c^*PL* phospholipids, *RBC* red blood cells

^d^Data are the mean ± SD without annotation

^e^*nd* not described

^f^Mean ± SE

^g^Mean

ARA supplementation does not result in decreased DHA/EPA composition as shown in Table 5. DHA/EPA composition was unchanged by 700 mg [70] or 838 mg [72] of ARA per day. In the same manner, 240 and 720 mg [71] or 1500 mg [73] of ARA per day did not change DHA/EPA composition. In contrast, it is well known that ARA composition is decreased by DHA/EPA supplementation [75, 76]. Interestingly, it is commonly observed that ARA supplementation results in large decreases in LA composition [71–74]. It appears that the capacity for exchange or retention in the body is in the following order DHA/EPA > ARA > LA. The substrate specificities of various acyl transfer reactions are thought to be related to this phenomenon; however, the details are unclear. It is important to consider the mechanism of LCPUFA metabolism, which requires further clarification.

## Conclusion

This review of dietary surveys of ARA intake indicates that ARA is obtained from a wide variety of animal foods, such as meat, poultry, egg, fish and dairy foods, and that the amount of ARA intake is 100–250 mg/day in advanced counties. Meanwhile, ARA intake may be in the tens of mg/day in developing countries. The review also demonstrates that ARA supplementation (82 or 120 mg/day for 3–4 weeks) at a dose equal to or less than the dietary ARA intake increases plasma ARA composition; that plasma ARA composition is ARA dose-dependently increased in the range of 82–3600 mg/day; and that ARA supplementation decreases plasma LA composition, but not DHA/EPA composition. ARA intake from foods or supplementation is thought to have a great impact on LCPUFA metabolism. The continued accumulation of evidence from large and well-designed dietary surveys and clinical trials is expected to confirm this.

## Abbreviations

ALA: α-linolenic acid
ARA: Arachidonic acid
DHA: Docosahexaenoic acid
DR: Dietary record
EPA: Eicosapentaenoic acid
FFQ: Food frequency questionnaire
LA: Linoleic acid
LCPUFA: Long-chain polyunsaturated fatty acids
PL: Phospholipids
RBC: Red blood cells
